# Using an abdominal phantom to teach urology residents ultrasound-guided percutaneous needle placement

**DOI:** 10.1590/S1677-5538.IBJU.2015.0481

**Published:** 2016

**Authors:** Pauline Filippou, Anobel Odisho, Krishna Ramaswamy, Manint Usawachintachit, Weiguo Hu, Jianxing Li, Thomas Chi

**Affiliations:** 1 Department of Urology, University of California, San Francisco, CA, USA; 2 Division of Urology, Department of Surgery, Faculty of Medicine, Chulalongkorn University, Bangkok, Thailand; 3 Beijing Tsinghua Changgung Hospital, Beijing, People's Republic of China

**Keywords:** Education, Percutaneous Nephrolithotomy, Ultrasonography

## Abstract

**Introduction:**

To assess the effect of a hands-on ultrasound training session to teach urologic trainees ultrasound-guided percutaneous needle placement.

**Materials and methods:**

University of California, San Francisco (UCSF) urology residents completed a time trial, placing a needle into a phantom model target under ultrasound guidance. Participants were randomized into three educational exposure groups: Group 1's time trial occurred prior to any teaching intervention, group 2's after experiencing a hands-on training module, and group 3's after exposure to both the training module and one-on-one attending feedback. Needle placement speed and accuracy as well as trainees' perceived confidence in utilizing ultrasound were measured.

**Results:**

The study cohort consisted of 15 resident trainees. Seven were randomized to group 1, three to group 2, and five to group 3. All residents reported minimal prior ultrasound experience. Their confidence in using ultrasound improved significantly after completing the training module with the most significant improvement seen among junior residents. Time to needle placement was fastest after receiving attending feedback (46.6sec in group 3 vs. 82.7sec in groups 1 and 2, p<0.01). Accuracy also improved with attending feedback, though the number of repositioning attempts did not differ significantly between groups.

**Conclusions:**

A hands-on training module and use of an abdominal phantom trainer increased resident confidence and skill in their use of ultrasound to guide percutaneous needle positioning. Attending feedback is critical for improving accuracy in needle guidance toward a target. Ultrasound-guided needle positioning is a teachable skill and can be applicable to multiple urologic procedures.

## INTRODUCTION

Percutaneous needle placement into the kidney is a skill of great utility for the practicing urologist. It is commonly used for percutaneous renal access to facilitate nephrostomy tube placement and percutaneous nephrolithotomy (PCNL) and also is applicable for renal biopsies and percutaneous ablation of renal masses (1, 2). All of these procedures are reliant on image-guided, accurate needle placement into different areas of the kidney. While ultrasound guidance is commonly used by interventional radiologists and nephrologists to position needles into the kidney for nephrostomy tube placement, renal biopsy, and tumor ablation (3), urologists may be less familiar with using renal ultrasonography to guide procedures.

Applying ultrasound (US) -guided needle placement to renal access during PCNL has been shown to result in decreased overall radiation exposure for patients and providers, as well as decreased blood loss during the procedure compared to fluoroscopy (4). Distinguishing posterior from anterior calyces is also an easier task using ultrasonography compared to fluoroscopy given the orientation of the US probe relative to the renal collecting system (5). Despite these benefits, the majority of urologists in the United States and around the world do not utilize ultrasound to guide needle placement for renal access during PCNL. This is likely due in part to the minimal emphasis placed on teaching renal US imaging during residency training (6). The goal of this study was to demonstrate that US-guided needle placement is a teachable skill for urologic trainees. We examined trainee experience level with US use for urologic procedures and evaluated the effect of an easily implementable teaching module on residents´ accuracy and precision in guiding a needle toward a target under US guidance in a simulated learning environment using an abdominal phantom trainer.

## MATERIAL AND METHODS

Resident trainees post-graduate year (PGY) 1-6 who were current members of the University of California, San Francisco Department of Urology residency program in December 2014 formed the study cohort. All trainees consented to study participation. After consulting with the institutional Committee on Human Research, this study was deemed exempt from approval.

**Figure 1:**
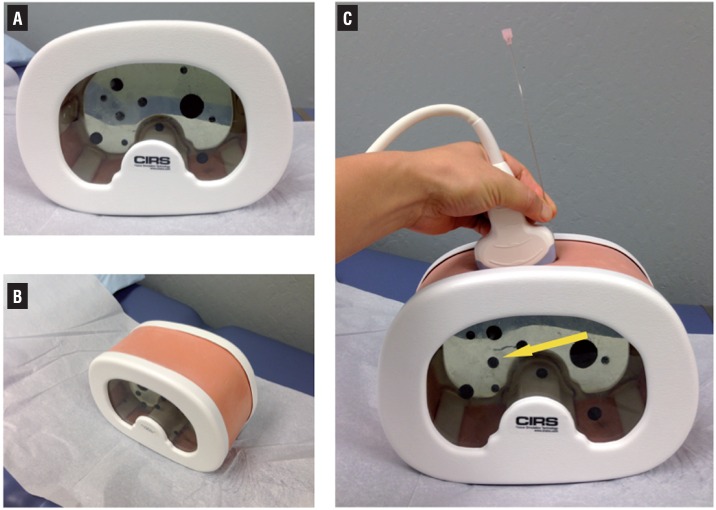
Abdominal training phantom used for ultrasound-guided needle placement time trial. The model has randomly distributed targets, visible in lateral (A) and oblique (B) views. Panel C demonstrates positioning of the ultrasound probe and needle used during the time trial. Participants were asked to place the needle into the black circular target indicated by the arrow.

**Figure 2:**
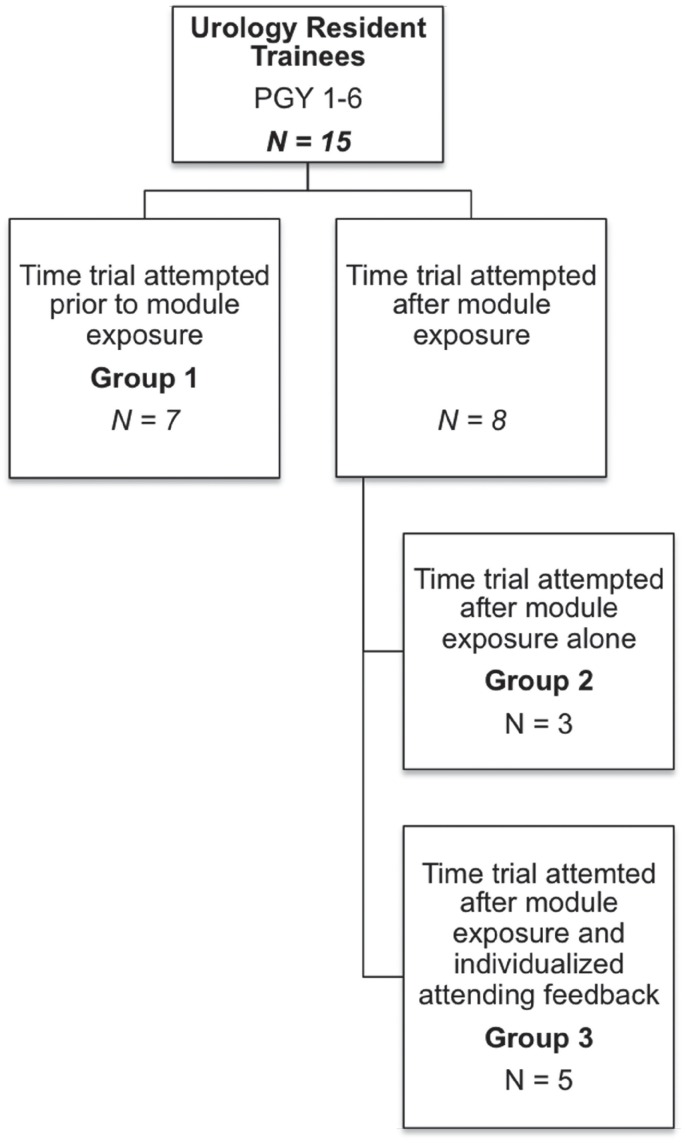
Description of UCSF urologic trainee cohort teaching exposures in each intervention arm.

Trainees were invited to participate in a training session and then randomized into three exposure groups. All participants were tasked with the objective of completing a time trial of placing a needle into a phantom target under US guidance (Figure-1). Group 1's time trial occurred prior to any intervention, group 2's time trial occurred after listening to a training module presented by an attending endourologist, and group 3's time trial occurred after exposure to both the training module and individualized attending feedback given during a hands-on experience with the US console and abdominal phantom (Figure-2). Participants in Groups 2 and 3 were both permitted to use the model for practice prior to their time trial, and Group 3 participants did so under the tutelage of an attending. For an effect size, we anticipated a three fold change in confidence level and two fold change in needle placement accuracy for participants in this study (7). Therefore, a sample size calculation dictated a minimal sample size of 4 participants in each exposure arm for this study design to achieve significance with a probability threshold of 0.05. Participants were randomized to each exposure group using an envelope randomization strategy. Opaque envelopes chosen by participants contained a paper marked with the word ‘before' or ‘after', determining whether or not the time trial would take place before or after module exposure. Furthermore, participants in the ‘after' group underwent secondary envelope randomization by the study administrators to receive attending feedback or not. Residents were overall anticipated to be mostly naïve to prior use of ultrasound-guided procedures and were therefore not block randomized by seniority. The training module provided a general overview of how renal US imaging is performed, different hand hold positions used for the US probe, how US guidance can be applied to renal tract access during PCNL, and several videos of renal percutaneous access performed under US guidance. These materials were designed by the authors to be practical and focused on skill acquisition. In addition, trainees had the opportunity to perform renal US imaging on one another as a part of this training module. Individualized attending feedback consisted of an endourology-focused attending experienced in renal US and US-guided needle placement providing instruction and immediate feedback to each trainee on the use of US while the trainee attempted to image and place a needle into a target in the phantom model. Two attendings provided ultrasound instruction. One (TC) who had recently begun performing PCNL under ultrasound guidance and one (JL) who had performed more than 8000 PCNL procedures under ultrasound guidance (8).

The phantom model (model 071A, CIRS, Inc., Norfolk, VA) is an abdominal US training phantom consisting of a housing containing 11 randomly positioned circular target lesions encased with a self-healing gel as well as a simulated spine and ribs. This phantom has been previously published and validated as a model for teaching image-guided biopsy technique (9).

The sizes of the targets embedded in the phantom range from 8-12mm, comparable to renal caliceal diameters in the moderately dilated collecting system. For this study, during the time trial, one of the medium-sized 10mm targets was pointed out to the trainee. The trainees were instructed to use US guidance to place a 15cm long, 18-gauge needle (Cook Echotip, Cook Medical, Bloomington, IN) into the indicated target (Figure-1). Time until the trainee felt the needle was placed into the intended target, number of needle repositioning attempts and needle placement accuracy (whether the phantom target was hit or not) were measured. Trainees took a survey before and after their training session which assessed trainee confidence in interpreting US images as well as using US to guide needle positioning during a procedure (Tables 1 and 2). Additional self-reported data regarding trainee characteristics (resident year, quantity of urologic procedures done under fluoroscopic guidance, and quantity of previous experience with US imaging) was also collected. Statistical analyses were performed using Student's t-test and Fisher's exact test using Stata 12.0 (Stata- Corp, College Station, TX).

**Table 1:** Trainee demographics and ultrasound experience.

Trainee characteristic	Value	Group 1	Group 2 and 3	P-value
		Teaching module taken after attempt (n=7)	Teaching module taken before attempt (n=8)	
Resident year, n(%)	Junior resident (PGY 1-3)	5(71)	3(38)	0.32
	Senior resident (PGY 4-6)	2(29)	5(62)	
Number of percutaneous renal procedures done using fluoroscopic guidance, n(%)	0-5	5(71)	2(24)	0.27
	6-10	0(0)	3(38)	
	>10	2(29)	3(38)	
Number of percutaneous renal procedures done under US guidance, n (%)	0-5	7(100)	8(100)	1.00
	>5	0(0)	0(0)	
Number of suprapubic tubes placed under US guidance, n(%)	0-5	6(86)	7(88)	1.00
	6-10	1(14)	1(12)	
	>10	0(0)	0(0)	
Number of times performing renal US for imaging. n(%)	0-5	6(86)	5(63)	0.71
	6-10	0(0)	2(25)	
	>10	1(14)	1(12)	
Number of times using US to image areas other than kidneys, n (%)	0-5	3(43)	2(25)	0.78
	6-10	1(14)	1(12)	
	>10	3(43)	5(63)	

**Table 2:** Trainee confidence scores (whole group) before and after ultrasound teaching module.

Confidence Level (Scale 1-10)	Value (N = 15)	Before teaching module	After teaching module	P-value
		mean (SD)	mean (SD)	
How confident do you feel that you could accurately interpret renal US imaging not performed by you (i.e. by a radiologist or technician)?	Junior resident	2.6(1.9)	5.3(1.7)	<0.01
	Senior resident	6.3 (3.0)	7.9 (0.9)	0.17
	All resident	4.3 (3.1)	6.5(1.9)	<0.01
How confident do you feel that you could accurately identify renal calyces using an US device?	Junior resident	3.3(1.5)	5.0(1.4)	0.01
	Senior resident	6.0 (2.2)	7.0 (1.5)	0.23
	All resident	4.5 (2.3)	5.9(1.8)	<0.01
How confident do you feel that you could accurately identify renal stones using an US device?	Junior resident	3.9(1.9)	5.9(1.9)	0.01
	Senior resident	5.4 (2.6)	7.0 (1.9)	0.03
	All resident	4.6 (2.3)	6.4(1.9)	<0.01
How confident do you feel that you could accurately place a needle into a target under US guidance?	Junior resident	2.3(1.9)	5.0(2.3)	0.06
	Senior resident	4.4 (2.6)	7.8 (1.6)	0.03
	All resident	3.3 (2.4)	6.3(2.4)	<0.01
Total confidence score (out of 40)	Junior resident	12.1(6.2)	21.1(6.4)	<0.01
	Senior resident	22.1 (9.8)	29.7 (4.1)	0.04
	All resident	16.8 (9.3)	25.1(6.9)	<0.01

## RESULTS

Fifteen trainees participated in this study; seven were randomized to group 1, three to group 2 and five to group 3. Neither the distribution of junior (PGY1-3) and senior (PGY4-6) residents nor trainee experience with US within group 1 differed significantly compared to groups 2 and 3. 100% of trainees reported doing less than five percutaneous renal procedures under US guidance during their training. 87% (N=13) of trainees reported using US for suprapubic tube placement less than five times. The majority of residents (73%, N=11) reported using US for renal imaging less than five times throughout their training. Residents reported the most experience with using US to image areas other than kidneys, with 53% (N=8) of residents reporting using US for this purpose over 20 times throughout their training (Table-1).

**Figure 3:**
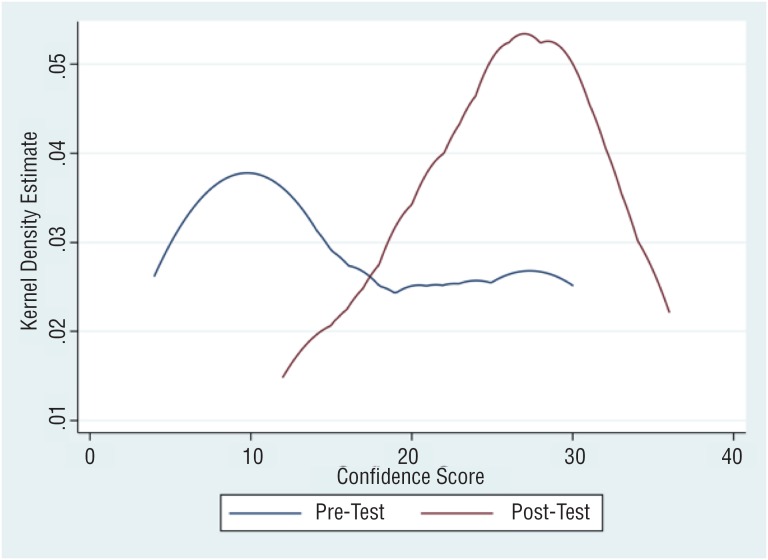
Kernel density distribution of total confi dence scores for trainees before and after ultrasound teaching module.

Perceived confidence in their ability to perform and interpret US imaging was compared amongst junior and senior level trainees, as well as amongst all participants before and after completion of their training session. Overall confidence scores of all participants improved after completing the training session (Figure-3). Total confidence scores (scale: 1-40; 40 = most confident) were significantly increased for all trainees after their training session (16.8 before vs. 25.1 after, p<0.01). This improvement was most significant for junior residents, PGY1-3 (12.1 vs. 21.1, p<0.01). Individual question scores (scale: 1-10; 10 = most confident) also showed significantly improved perceived confidence after completing the training module across a variety of skills, with residents feeling more confident in interpreting renal imaging (6.5 after vs. 4.3 before, p<0.01), identifying renal calyces (5.9 vs. 4.5, p<0.01), identifying renal stones (6.4 vs. 4.6, p<0.01), and accurately placing a needle into a target under US guidance (6.3 vs. 3.3, p<0.01). Senior residents, PGY4-6, felt improved confidence in their ability to accurately place a needle into a target under US guidance (7.8 vs. 4.4, p = 0.03) whereas junior residents felt statistically significantly improved confidence in all question areas except for this one (5.0 vs. 2.4, p = 0.06). Senior residents felt more confident interpreting renal US imaging not done by them and identifying renal calyces following completion of the training module, though changes in these question scores were not statistically significantly different (Table-2).

When comparing accuracy of placing a needle into a phantom target under US guidance during a time trial among participants, time to perceived target acquisition, number of attempts utilized and whether the needle actually landed in the target improved the most for group 3, who received one-on-one training with attending feedback prior to their time trial (Table-3). Time to perceived needle placement was significantly faster after attending feedback was given (46.6sec in group 3 vs. 82.4sec in groups 1 and 2, p = 0.04). The number of needle attempts needed to reach the target decreased following attending feedback, though this difference did not reach statistical significance (4.0 needle attempts vs. 6.9, p=0.28). In addition, the percentage of needles successfully placed into the target increased following individualized attending feedback, though this difference was not statistically significant (80% success vs. 43% success, p=0.36).

**Table 3:** Univariate analysis of needle placement accuracy comparing trainees who made their attempt before or after being exposed to ultrasound teaching module and attending feedback.

Accuracy		Group 1 and 2 n = 10	Group 3 n = 5	P value
Time to target in seconds, mean (SD)		82.7 (32.1)	46.6 (21.5)	0.04
Number of attempts, mean (SD)		6.9 (5.4)	4.0 (2.6)	0.28
Needle successfully placed in target, n (%)	Yes	4 (43)	4 (80)	0.36
	No	6 (57)	1 (20)	

## DISCUSSION

Renal ultrasound is a tool used to facilitate percutaneous renal procedures. These include obtaining percutaneous renal access for PCNL and nephrostomy tube placement (10), performing percutaneous renal biopsies (11), and placement of needles for focal lesion ablation (1). All of these procedures rely on accurate use of imaging to guide needle placement within the kidney into the desired location. Ultrasonography is an ideal imaging platform for needle guidance and a simulation environment provides a means for trainees to increase their familiarity with ultrasound techniques in a safe, low pressure learning environment (7). Our study demonstrates that with the use of an abdominal phantom, resident trainees can garner confidence and acquire skills to support their clinical training for ultrasound-guided percutaneous procedures.

The use of PCNL is considered the minimally invasive standard of care for surgically removing large renal stones (12, 13). There are several technical challenges for the practicing urologist to overcome in order to perform this procedure effectively. Precise puncture into the appropriate calyx is paramount, and achieving successful collecting system needle access has been quantified as significantly contributing to the steep learning curve attributed to PCNL. De la Rosette et al. quantified the learning curve for gaining access during PCNL utilizing fluoroscopic guidance, and recommended a minimum of 24 PCNLs during training in order to attain good surgical proficiency, and a total of 60 to achieve competency (14). Current Accreditation Council for Graduate Medical Education (ACGME) urologic residency training standards require a minimum of ten percutaneous renal endourology procedures performed in order to successfully complete residency training (15). Thus, reaching the minimum number of PCNL procedures residents are required to perform during residency to meet ACGME competency requirements may not be sufficient for proficiency in gaining percutaneous renal access for this procedure by a graduating urologist, much less utilizing US to gain access.

Along with the overall infrequent use of and training in US needle guidance during PCNL, another disadvantage for training urologists in this modality lies in the nature of the overall procedure itself. What has been quantified as the most difficult and arguably most important part of the procedure to learn-gaining access into the appropriate calyx of the kidney-is only done once per PCNL in the absence of the need for multiple access tracts. Thus, training opportunities in the operating room are naturally diminished by the nature of the procedure.

US has been shown in previous studies to be an excellent adjunct tool to aid in gaining percutaneous access into the collecting system (16). Using US guidance for this purpose allows visualization of the vasculature of the kidney, which can contribute to decreased blood loss during PCNL (5). Additionally, the harms of significant ionizing radiation exposure to the patient and providers are decreased when using US-guided access during training. Jag-tap et al. found that trainees utilizing US guided access during PCNL had less radiation exposure during renal access than those using fluoroscopy alone (17). Despite the benefits of US guidance, this study shows that urologic residents at our institution are not receiving significant exposure to using renal US as an imaging tool during their training. This experience is likely reflected across most training programs in the United States.

Several studies have shown that the use of virtual reality simulators improves urology trainee skills in areas such as endoscopy and robotics, but the use of US trainers as a part of urologic education has not been thoroughly evaluated (18). This study demonstrates the effect of a teaching module and attending feedback on the US skills and perceived confidence levels of urology residents. Our results highlight the fact that US imaging and needle guidance are skills that can be effectively taught to trainees through hands-on training during residency with tangible improvement in resident skill and confidence.

We examined hands-on simulator training as an alternative to in vivo experience for the training of urologic residents in the skill of needle positioning and guidance with US. This training session only required one hour of time for each trainee, incorporating visual information on how to use US, a brief didactic lecture, and a hands on experience, but was found to significantly improve resident confidence scores in using US as a tool to aid in target access, as well as significantly improve confidence in interpreting US. This improvement was particularly significant among junior residents, suggesting that this type of education may be most useful if done early in urologic training.

When looking at the effect of this training session on US skill, one-on-one attending teaching and feedback was found to be the most critical piece for improving trainee accuracy in being able to guide a needle toward a target. Though some improvement was seen in the group who received the education module before attempting their time trial compared to those who did not receive the module before the attempt, the most significant improvement in time to reaching the target occurred in the group that received attending feedback and teaching. Improved confidence and skill acquisition support trainees to move forward with continuing to perform procedures when they leave the training environment (7, 11). Teaching interventions like the one described in our study that not only facilitate skill acquisition but also improve the learner's confidence may therefore translate to improved procedure performance in a real world setting. This is particularly relevant for the teaching of percutaneous nephrolithotomy, the procedure most commonly performed by urologists to which percutaneous renal needle positioning is applicable.

One major limitation for our study was the small overall group and subgroup sizes. Based on our sample size calculations, since a large effect size was anticipated, an adequate number of participants were enrolled to detect differences in confidence and skill levels between groups. Despite this, the small sample size may have decreased our power to detect differences in performance between groups that may otherwise not be readily apparent. Accuracy and number of needle placement attempts improved with attending feedback, though not significantly. This difference in significance may have been confounded by the small sample size. Also, due to the small sample size, no multivariate analysis of the effect of the teaching module on resident skill could be performed to control for factors such as resident experience level or quantity of US experience. Despite limited sample sizes, however, others have validated the effects of simulated training on resident learning with studies where small sample sizes were utilized (19, 20). Therefore, while our sample size was small, it was not outside of a reasonable range to be expected for this type of study.

In addition, this study evaluated the experience and confidence levels within a single residency training program, one in which urology residents have had very little prior exposure to the use of ultrasound. To date, the majority of PCNL procedures had been done under fluoroscopic guidance at our program, and this cohort of residents therefore had little training in the use of ultrasonography outside of transrectal ultrasound. These study results thus focus on urologists who are early in there training, but already committed to urology. Ideally multiple institutions could be surveyed in the future for a more in-depth look at training in US-guided renal access to gain a more generalizable understanding of how urology residents receive training in the United States. Future studies might also include medical students as well as more experienced urologists to see if these types of simulator training sessions have similar effects on trainees of different levels.

We also utilized a non-validated set of questionnaires to evaluate trainee confidence levels. Specific to the tasks examined in our study, no validated questionnaires exist, leading us to create a set of questions that were resultant of a discussion between members of the research team. These questionnaires were intended to capture trainee confidence and experience and query their experiences related to ultrasound and percutaneous needle placement but could be made to be more comprehensive and generalizable during future studies. Based on our study results, appropriately powered studies across multiple institutions could be planned to validate and confirm our findings.

Lastly, this study looked at the effect of a training session on the ability of participants to place a needle into a target within an abdominal phantom and on their perceived confidence in using US. For a urologist in training, clinical performance in real-life situations is the ultimate measure of how impactful a training session is for their overall ability to care for patients. While our study demonstrated that participants performed better on a needle placement task in a training environment and felt that they could better image the kidney in future settings, we did not measure the impact of this training session on actual clinical performance. Several challenging factors are inevitably present in real patients that are difficult to simulate in the training environment when relying on phantoms as training models. These include the movement of the kidney with respiration, the variation of kidney depth relative to body habitus, and the presence of small caliceal puncture targets in the context of the non-dilated collecting system. Our phantom facilitated a training environment that provides the trainee with tools to learn ultrasound guided needle placement, but these more complex situations were not simulated with our training phantom.

In clinical practice, to overcome some of these complex situations, needle guides are sometimes used to increase the accuracy of needle placement. These are particularly popular during percutaneous renal biopsy procedures. In practice, our clinical team routinely performs percutaneous renal access without a needle guide, but recognizes that their use is certainly not unreasonable. However, needle guides can be limiting in some circumstances where the angle of entry needed for the needle to enter the skin and the kidney lies outside of what the needle guide permits. For example, if the patient's kidney has a particularly sharp infundibulopelvic angle, it might be advantageous to enter the kidney with a very shallow angulation relative to the skin. A needle guide might not facilitate this angle of entry, despite some guides having multiple possible positioning angles available for use. Therefore, in order for a trainee to apply ultrasound guidance to any variety of clinically relevant scenarios, we feel that the most critical skill for the trainee to acquire is the coordinated ability to track movement of the needle under real time ultrasound imaging.

To this end, the clinical relevance of our results warrants particular consideration as the study was performed in a simulated environment. Our results demonstrate that trainees can learn how to image a needle and coordinate their hands so that they can guide needle placement toward a target and that their confidence in this skill improves with a single, relatively easily implemented teaching encounter. We think that these results have clinical relevance from several perspectives. Our study demonstrated that the physical skill of ultrasound guided needle placement is learnable. Performing this feat relies on two technical skills. First, the imaging hand must maintain a steady image of the target and the desired path to that target. Second, the needle hand must advance the needle within the imaging plane in order to achieve needle insertion into the target. While these are the only two technical skills requiring mastery in order to learn ultrasound guided needle access, they rely on two hands gaining independent skills and then coordinating those skills between the two hands and the visual image seen on the screen. A simulated environment is therefore an ideal arena in which to acquire these technical skills, without which, needle guidance in a clinical setting would not be easy to accomplish. Our study focused on the acquisition of these technical skills, and therefore lays the foundation for clinical translation of these skills to procedural use. In addition, literature supports the idea that if trainees feel more confident in performing a skill, they are more likely to continue utilizing that skill after completing their training (7). In today's practice environment, the majority of urologists do not obtain their own percutaneous renal access for PCNL (6). We contend that if ultrasound needle guidance can be taught in a manner that is adoptable and facilitates urologic trainee's confidence in that skill set, they may be more likely to obtain their own renal access or perform percutaneous ultrasound-guided procedures in practice after completing their training. We hope that this might eventually lead to urologists obtaining their own percutaneous renal access for PCNL, which might facilitate safer procedures in the future. This current study, centered around teaching of the technical skill itself, was limited in our ability to evaluate these latter hypotheses. However, it lays the groundwork to future studies that will show the relevance of simulated ultrasound guided needle placement training to clinical practice, including both the urologist's ability to apply these skills to clinical practice as well as the adoption of these skills into their routinely used arsenal of patient care tools.

Ultimately, the goal of this present study was to demonstrate the concept that ultrasound guidance for needle placement, which could also be applied to percutaneous renal mass biopsy and ablation as well as percutaneous nephrolithotomy, is an easily teachable skill for urologic trainees. Indeed, these training sessions generated increased confidence and enthusiasm for these skills within the participating trainees, and these are effects that we hope will carry over into future aspects of each resident's training and clinical practice. We consider these present results as early steps toward more widespread adoption of ultrasound training for urology residents. Based on our current findings, we believe that ultrasound needle guidance is a teachable, learnable skill. We plan that future studies will expand on whether these simulated skills translate to application in a real world procedural setting.

## CONCLUSIONS

This educational exposure study shows that a short, formalized training session in US use can be readily implemented in urologic training to improve resident skill and confidence. US-guided percutaneous needle positioning is a teachable, achievable skill that is effectively taught with a combination of didactics, hands-on training, and, most importantly, one-on-one attending feedback.
